# Self-control, future orientation, smoking, and the impact of Dutch tobacco control measures

**DOI:** 10.1016/j.abrep.2015.07.002

**Published:** 2015-07-29

**Authors:** Michael Daly, Liam Delaney, Roy F. Baumeister

**Affiliations:** a Behavioural Science Centre, Stirling Management School, University of Stirling, Scotland, United Kingdom; b UCD Geary Institute, University College Dublin, Ireland; c Department of Psychology, Florida State University, Tallahassee, FL, United States

**Keywords:** Self-control, Future orientation, Smoking, Smoking restrictions, Tobacco control

## Abstract

**Introduction:**

The pronounced discrepancy between smokers' intentions to quit and their smoking behavior has led researchers to suggest that many smokers are time inconsistent, have self-control problems, and may benefit from external efforts to constrain their consumption. This study aims to test whether self-control and future orientation predict smoking levels and to identify if these traits modify how cigarette consumption responds to the introduction of tobacco control measures.

**Methods:**

A sample of Dutch adults (N = 1585) completed a measure of self-control and the Consideration of Future Consequences Scale (CFCS) in 2001 and indicated their tobacco consumption each year from 2001 to 2007. In 2004, a workplace smoking ban and substantial tax increase on tobacco was introduced in the Netherlands. To identify the potential impact of these tobacco control measures we examined whether participants smoked or were heavy smokers (20 + cigarettes per day) each year from 2001 to 2007.

**Results:**

Participants with high self-control and CFCS scores showed lower rates of smoking across the seven year period of the study. The 2004 smoking restrictions were linked with a subsequent decline in heavy smoking. This decline was moderated by self-control levels. Those with low self-control showed a large reduction in heavy smoking whereas those with high self-control did not. The effects were, however, temporary: many people with low self-control resumed heavy smoking 2–3 years after the introduction of the tobacco restrictions.

**Conclusions:**

The immediate costs which national tobacco control measures impose on smokers may assist smokers with poor self-control in reducing their cigarette consumption.

## Highlights

• Shows that self-controlled and future orientated adults have a low rate of current smoking
• Links major 2004 Dutch tobacco control measures to a reduction in heavy smoking
• Demonstrates that this reduction in heavy smoking was greatest for the less self-controlled

## 1 Introduction

Tobacco use is the largest lifestyle contributor to health conditions globally and there is currently strong support for an accelerated implementation of national tobacco control strategies (Beaglehole et al., 2011, Danaei et al., 2009, Jha and Chaloupka, 1999). A body of research exists detailing how psychological characteristics may affect the prevalence of smoking. Smokers appear to be less future oriented (Adams and Nettle, 2009, Reynolds et al., 2004) and to have lower self-control than others (Daly et al., 2015, Muraven, 2010a). In the current study we examine how these psychological characteristics relate to tobacco consumption and test whether future orientation and self-control produce heterogeneity in how cigarette use responds to large scale tobacco control measures.

### 1.1 Time perspective, self-control, and smoking

Government health campaigns often promote the long-term benefits of forgoing tempting behaviors (e.g. smoking, alcohol consumption), taking preventative action (e.g. undergoing screening, health regular checks), and investing consistently in protective behaviors (e.g. exercise, healthy diet). Those who are future oriented, as assessed using measures of time perspective (e.g. Strathman, Gleicher, Boninger, & Edwards, 1994), show lower levels of smoking, alcohol consumption, and body mass index than others (Adams and Nettle, 2009, Adams and White, 2009, Beek et al., 2013, Delaney et al., 2008). Of the many health behaviors, there is a particularly strong rationale for linking time perspective, self-control and tobacco consumption. Smokers are acutely aware of the financial impact of tobacco use and largely recognize the negative health effects of smoking (Hammar & Carlsson, 2005). Furthermore, most smokers wish to quit and try to do so regularly (Lader, 2007).

The strong cessation goals that characterize smokers suggest that their behavior represents a self-control problem: smokers intend to smoke less, yet the short-term benefits of smoking (e.g. pleasurable experience, avoidance of withdrawal symptoms) undermine this long-term goal (Adams, 2009). Self-control is conceptually related to time perspective, but empirical studies have shown only modest relations between widely used measures of both concepts (e.g. Adams and Nettle, 2009, Daly et al., 2009). Like those with a low level of future orientation, those with poor self-control are prone to smoking (Daly et al., 2014, Daly et al., 2015, Moffitt et al., 2011) and training to effectively increase self-control (e.g. Muraven, 2010b, Muraven et al., 1999) can assist smokers in reducing their consumption and successfully quitting (Muraven, 2010a, Oaten and Cheng, 2006).

While markedly enhancing self-control is difficult and involves a considerable commitment on the part of the individual, externally imposed restrictions may partially diminish the necessity for self-control and attenuate the impact of low future orientation. Those who are particularly time-sensitive to rewards in the immediate rather than the distant future (Laibson, 1997) may even want restrictions placed upon their behavior in order to act in a way that maximizes long-term rather than immediate benefits. For instance, smokers who want to quit are more supportive of smoking restrictions and tax increases than other smokers (Kan, 2007). There is even evidence that the well-being of those who have a propensity to smoke may improve after taxation is placed on tobacco products (Gruber & Mullainathan, 2005). Similarly, the introduction of a smoking ban appears to increase the well-being of those who have recently failed to give up smoking, suggesting that such bans may assist smokers in following their ideal consumption pattern (Odermatt & Stutzer, 2013).

In these cases, the imposition of a tax creates a restriction on a behavior which has had damaging effects on health and well-being. Restricting smoking and increasing tax on tobacco products places an immediate cost on smoking that could enhance the ability of those with low future orientation and self-control to reduce their smoking levels. In this study, we therefore examine how these psychological characteristics interact with the introduction of stringent tobacco control measures in the Netherlands in 2004.

### 1.2 Tobacco control measures in the Netherlands

On January 1st 2004 a workplace smoking ban, from which the hospitality industry was exempt, was implemented in the Netherlands. This was followed by a large tax increase of 20% on February 1st 2004. The beneficial health effects of these tobacco control measures have been documented extensively (e.g. de Korte-de Boer et al., 2012, Verdonk-Kleinjan et al., 2009). Nagelhout, Willemson, and de Vries (2011) used data from 2001 to 2007 drawn from the Dutch Continuous Survey of Smoking Habits (DCSSH) to show an increase in quit attempts and a decrease in the prevalence of smoking in the first half of 2004. Using the DCSSH, Verdonk-Kleinjan, Candel, Knibbe, Willemsen, and de Vries (2011) showed that the workplace ban alone did not produce a decline in smoking levels but the ban coupled with the later 20% tax increase reduced the prevalence of daily smoking and the number of cigarettes smoked per day amongst participants in paid work.

The present study used a sample of Dutch adults to test the hypothesis that individual differences in self-control and considerations of future consequences are associated with the prevalence of smoking and heavy smoking. Furthermore, to gauge how tobacco consumption may be affected by national smoking regulations, we examined the change in smoking levels following the introduction of the 2004 workplace smoking ban and subsequent 20 per cent tax increase in the Netherlands. Finally, we tested whether changes in smoking behavior following these national interventions varied as a function of two personality traits: self-control and future orientation.

## 2 Method

### 2.1 Participants and procedure

Data were drawn from the Dutch National Bank Household Panel (DHP), an annual representative survey of the Dutch population aged 16 and over. The DHP data were collected through the CentERpanel, an internet-based survey panel of 2000 Dutch households. To ensure representativeness, a television and telephone line based internet system was provided to all participating households lacking a personal computer with an internet connection. The survey includes eight central modules administered each year (see http://www.centerdata.nl), and the sampling methods including details on response rates and sampling bias have been documented extensively (Nyhus, 1996). In total, 1585 participants provided basic demographic details and information on their smoking levels as part of the 2001 survey and the characteristics of the sample are detailed in Table 1. The average age of those who took part in the 2001 survey was 45.17 (SD = 13.67), 44.4% were female, 11.2% had a disability, and the average household size was 2.66 (SD = 1.36).

**Table 1:** Descriptive statistics for main study variables and demographic characteristics in 2001.

Variable	N	Mean/%	SD
*Psychological variables*			
Self-control	1060	5.20	1.07
Consideration of Future Consequences Scale (CFCS)	1218	44.60	7.29

*Health behavior*			
Smoker (%)	1585	29.40	
Smoke 20 or more per day (%)	1585	9.78	

*Demographic factors*			
Age	1585	45.17	13.67
Female (%)	1585	44.35	
Education level completed^a^	1585	5.38	2.53
Income	1585	40,539.46	29,092.12
Employed (%)	1585	62.97	
Unemployed (%)	1585	1.31	
Retired (%)	1585	12.30	
Disabled (%)	1585	11.17	
Other (%)	1585	12.24	
Household size	1585	2.66	1.36
Level of urbanization^b^	1585	2.83	1.32

a 0 = not yet attending any education; special (low-level) education; other sort of education/training/ apprenticeship, 1 = kindergarten/primary, 2 = continued primary education or elementary secondary education, 3 = continued special (low-level) education, 4 = pre-university education, 5 = junior vocational training, 6 = senior vocational training, 7 = vocational colleges, 8 = vocational colleges 2nd tier, and 9 = university education.

b From 1 = very high degree of urbanization to 5 = very low degree of urbanization.

In order to identify the impact of the psychological characteristics examined on subsequent smoking levels our analyses use personality data from the ‘economic and psychological concepts’ section of the 2001 survey. Those with available data on relevant covariates who provided self-control data (N = 1060) did not differ from those who did not complete this section, either in terms of demographic or background characteristics. Participants who completed the Consideration of Future Consequences Scale (CFCS) (N = 1218) were likely to be older, have higher income, be retired or disabled and live in lower density areas, providing some evidence of selective completion of this measure.

The current study utilized all waves of data from 2001 to 2007, thus spanning an extensive period prior to and after the introduction of major changes in tobacco control in the Netherlands in early 2004. Approximately 56% of participants with self-control data and 50.5% of those with CFCS data dropped out of the sample between 2001 and 2007, a rate of attrition of 7.2%–8% per annum. An examination of potential attrition bias in the key study variables showed that those who dropped out did not appear to have different levels of self-control from those retained in the sample. For example, the self-control levels for those retained in the sample differed little across the seven years examined (Min M = 5.20, SD = 1.07; Max M = 5.26, SD = 1.04).

Similarly, CFCS scores appeared to differ minimally across the seven years of data utilized (Min M = 44.52, SD = 7.45; Max M = 44.76, SD = 7.39). Thus, the analyses failed to identify evidence of attrition bias where participants dropped out of the sample non-randomly as a function of their pre-existing self-control or CFCS levels. In total, 4528 observations of smoking behavior were obtained from the seven years of data provided by the 1060 participants who completed the self-control measure in 2001. For the portion of the sample that provided information detailing the extent to which they consider the future (N = 1218) 5631 smoking status observations were recorded between 2001 and 2007 and included in the analyses.

### 2.2 Measures

#### 2.2.1 Self-control

The self-control item used in the current study was drawn from an abbreviated measure of the 16 Personality Factors (Cattell, Eber, & Tatsuoka, 1970), known as the 16 Personality Adjectives (Brandstatter, 1988). Participants were asked to describe their personality using pairs of opposing personal qualities. Self-control was measured using a single item where participants rated their level of self-control on a scale from 1 (*little self-control*) to 7 (*disciplined*). Participants tended to rate themselves as self-controlled, as indicated by a mean score of 5.20 (SD = 1.07).

Self-control scores were analyzed as continuous in all multivariate analyses. We grouped self-control scores into three groups, approximately representing those 1 SD below and above mean self-control levels (M = 5.2, SD = 1.07), for the purpose of graphing results and interpreting interaction effects. Those who provided ratings from 1 to 4 (24.1% of the sample) were deemed to have low self-control, ratings of 5 were labeled medium self-control (29.9%), and scores of 6–7 were considered indicative of high self-control (46%).

#### 2.2.2 Consideration of Future Consequences Scale

The CFCS (Strathman et al., 1994) was administered to participants in 2001. Participants rated each item on a 7-point scale from 1 (*disagree strongly*) to 7 (*agree strongly*). For unknown reasons, only 11 of the original 12 CFCS items were administered in the DHP. On average participants rated themselves to be approximately at the mid-point on the CFCS (M = 44.60, SD = 7.29; Min = 19, Max = 74), and the scale showed an acceptable level of reliability (Cronbach's alpha = .75). The CFCS was treated as a continuous variable in all analyses. We conducted sensitivity analyses to gauge the potential impact of the missing CFCS item (“Since my day to day work has specific outcomes, it is more important to me than behavior that has distant outcomes.”) on our results. To do this we utilized a sample of 198 students (mean age = 23.3 (SD = 6.1), 66% female) recruited as part of a Day Reconstruction Method study (described in Daly, Delaney, Doran, Harmon, & MacLachlan, 2010). Our analyses showed that excluding the missing item did not affect the reliability of the CFCS (Cronbach's alpha value for 12-item CFC = .80; for 11-item CFC = .80) or the average item score on the scale. The correlation between the CFCS and whether the participant was a smoker (14.1% of the sample) was also relatively unchanged by the exclusion of this item (12-item CFCS: *r* = −.23, p < .001, 11-item CFCS: *r* = −.25, p < .001). Our sensitivity analyses therefore indicated that the absence of the 12^th^ CFCS item was unlikely to have markedly affected our results.

#### 2.2.3 Smoking

Each year from 2001 to 2007 participants were asked “*Do you smoke cigarettes at all?*” A dichotomous variable was formed where those who selected the options “*Yes, I smoke every day*” or “*Yes, I smoke every now and then*” were categorized as smokers. In total, 29.4% of the sample identified themselves as smokers, which was below the figure for smoking levels identified in the Annual Statistics Netherlands Health Survey in 2001, which was 34.7% (Statistics Netherlands, 2012). Smokers were then asked “*About how many cigarettes do you smoke a day?*” and responded using the two response categories provided: ‘*less than 20 cigarettes a day*’ or ‘*at least 20 cigarettes a day*’. Those who indicated they smoke at least 20 cigarettes per day were deemed to be heavy smokers. In total, 9.8% of participants were heavy smokers, once again slightly below the figure identified by Statistics Netherlands in 2001, which was 10.4% (Statistics Netherlands, 2012). Thus, whilst a large portion of the sample were smokers and many smoked heavily, it does appear that smoking levels may have been underestimated by approximately 10% when contrasted with those of the Dutch population.

#### 2.2.4 Demographic and background characteristics

Age, gender, socioeconomic status, occupation, household size, and urban density of the area where the participant resides were assessed in 2001 and included as covariates in all analyses. Net annual income and education were utilized as the main markers of socioeconomic status. The education variable was analyzed as a continuous variable where nine categories represented various levels of progression through the education system ranging from 0 (*not yet attending any education; special (low-level) education; other sort of education/training/apprenticeship*) to 9 (*completion of university degree*). Current occupation was classified into ‘employed’, ‘unemployed’, ‘retired’, ‘disabled’ and ‘other’ categories as shown in Table 1. Household size was equal to the total number of children and adults living in the household at the time of the survey. Finally, participants rated the degree of urbanization of their area of residence on a scale from 1 (*very high degree of urbanization*) to 5 (*very low degree of urbanization*).

### 2.3 Statistical analyses

Due to the hierarchical nature of the data, with repeated measures of smoking behavior (Level 1) nested within individuals (Level 2), generalized estimating equations (GEE) models with a probit link function and exchangeable correlation matrix structure (assuming homogenous correlations between elements over successive measurements) were used (e.g. Heck, Thomas, & Tabata, 2013). GEE can be applied when there is an uneven number of assessments for each participant, as was the case in the current study where some participants dropped out of the panel immediately and others were retained for several years. It also allows adjustment to be made for autocorrelation between repeated observations within each participant.

In the current study two outcome variables were examined: whether the participant is a smoker, and whether the participant smokes heavily (20 + cigarettes per day). We estimate separate random coefficient analyses for each outcome. The initial analyses tested whether between-person differences in personality (Level 2) predicted smoking behavior (Level 1). To clearly specify the relationship between personality and smoking we adjust for a broad set of covariates including the linear pattern of change in smoking over time and individual level demographic and background characteristics, as shown in *model 1* below. In the standard nomenclature employed, *t* represents the survey year, and *i* represents the individual.

Following this, we sought to test whether smoking levels declined markedly after the introduction of a set of regulatory changes in early 2004. To do this, a dichotomous variable was formed which identified whether the wave examined was before or after the introduction of the tobacco control measures (0 = 2001–2003 and 1 = 2004–2007). This variable was used to predict smoking levels adjusting for the linear effect of time and demographic and background factors, as outlined in *model 2*.

We then aimed to test whether individual differences in personality traits, self-control and consideration of future consequences, modified the extent to which smoking behavior changed after tobacco control measures were introduced in the Netherlands. This analysis tests whether the effect of a level 1 variable (smoking restrictions) varies across scores on a level 2 variable (personality). This was achieved by including an interaction term between the personality traits examined and the dichotomous variable indicating whether each wave was pre- or post-smoking restrictions, as detailed in *model 3* below. Finally, to formally identify how the slope of the relationship between tobacco control measures and smoking levels differed as a function of personality, we regressed smoking restrictions on smoking behavior at low and high levels of the personality trait examined.

Model 1
  Level 1: Smoking levels_*ti*_ = *β*_0*i*_ + *β*_1*i*_(Year_*ti*_) + *r*_*ti*_
  Level 2: *β*_0*i*_ = γ_00_ + γ_01_(Personality_*i*_) + γ_02_(Demographics/Background_*i*_) + *u*_0*i*_
Model 2
  Level 1: Smoking levels_*ti*_ = *β*_0j_ + *β*_1*i*_(Year_*ti*_) + *β*_2*i*_(Tobacco Control Measures_*ti*_) + *r*_*ti*_
  Level 2: *β*_0*i*_ = γ_00_ + γ_01_(Demographics/Background_*i*_) + *u*_0*i*_
Model 3
  Level 1: Smoking levels_*ti*_ = *β*_0*i*_ + *β*_1*i*_(Year_*ti*_) + *β*_2*i*_(Tobacco Control Measures_*ti*_) + *r*_*ti*_
  Level 2: *β*_0*i*_ = γ_00_ + γ_01_(Personality_*i*_) + γ_02_(Demographics/Background_*i*_) + *u*_0*i*_*β*_2*i*_ = γ_20_ + γ_21_(Personality_*i*_) + *u*_2*i*_

## 3 Results

### 3.1 Descriptive analyses

Self-control scores showed a weak positive relationship with CFCS scores (r = .08, p < .01), as shown in Table 2. Older and better educated participants and those on higher incomes had higher levels of self-control than others. Consideration of the future was greatest amongst young people and those with high levels of education and income. Highly educated individuals and those on high incomes were also less likely than others to smoke as is typical (Cutler & Lleras-Muney, 2010).

**Table 2:** Correlation matrix detailing relationships between key study variables.

	Self-control	CFCS	Smoke	Smoke 20 +	Age	Fem.	Income	Educ.	HH size
CFCS	.08⁎								
Smoke	−.13⁎⁎	−.09⁎⁎							
Smoke 20 plus	−.12⁎⁎	−.05	.51⁎⁎						
Age	.07⁎	−.09⁎⁎	−.08⁎⁎	−.02					
Female	−.07⁎	−.06⁎	.02	.02	−.11⁎⁎				
Income	.14⁎⁎	.11⁎⁎	−.06⁎	−.03	.14⁎⁎	−.45⁎⁎			
Education level^a^	.17⁎⁎	.14⁎⁎	−.06⁎	−.07⁎⁎	−.16⁎⁎	−.09⁎⁎	.27⁎⁎		
Household size	−.08⁎⁎	.03	−.05	.00	−.25⁎⁎	.05	−.08⁎⁎	.00	
Urbanization^b^	.02	−.03	−.07⁎⁎	−.07⁎⁎	.03	.01	−.08⁎⁎	−.03	.25⁎⁎

a 0 = not yet attending any education; special (low-level) education; other sort of education/training/apprenticeship, 1 = kindergarten/primary, 2 = continued primary education or elementary secondary education, 3 = continued special (low-level) education, 4 = pre-university education, 5 = junior vocational training, 6 = senior vocational training, 7 = vocational colleges, 8 = vocational colleges 2nd tier, and 9 = university education.

b From 1 = very high degree of urbanization to 5 = very low degree of urbanization.

⁎⁎ p < 0.01.

⁎ p < 0.05.

### 3.2 Self-control and smoking levels

An examination of the bivariate correlations showed that self-control was negatively related to smoking status (r = −.13, p < .01). In total, 38% of those with low self-control were smokers, in contrast to just 22.8% of participants with high self-control. Self-control scores were also negatively correlated with heavy smoking (r = −.12, p < .01). Our GEE model test of *model 1* showed that self-control scores were inversely related to the likelihood of smoking (*b* = −.13, SE = .04, χ^2^ = 12.77, p < .01) in analyses which adjusted for a large set of demographic and background characteristics (age, gender, education, labor force status, household size, urbanization). Similarly, the self-controlled had a significantly lower likelihood of smoking 20 or more cigarettes per day, (*b* = −.11, SE = .05, χ^2^ = 5.47, p < .05) in fully adjusted analyses, as detailed in Table 3. Our analyses also showed a decline in the prevalence of smoking (*b* = −.03, SE = .01, χ^2^ = 16.04, p = .01) and heavy smoking (*b* = −.03, SE = .01, χ^2^ = 9.58, p < .01) from 2001 to 2007.

**Table 3:** Results of generalized estimating equations models assessing the relationship between self-control and both smoking status and high cigarette consumption (N = 1060).

Variable	Smoking status			High cigarette consumption		
	B	SE	χ^2^	B	SE	χ^2^
Year	−.03	.01	16.04⁎⁎	−.03	.01	9.58⁎⁎
Self-control	−.13	.04	12.77⁎⁎	−.11	.05	5.47⁎
Age	−.00	.00	1.41	−.00	.00	.03
Female	−.05	.09	.24	−.06	.12	.25
Education level completed^a^	−.03	.02	3.73	−.05	.02	5.04⁎
Income	.00	.00	2.06	.00	.00	.19
Unemployed (%)	.77	.31	6.18⁎⁎	.74	.36	4.30⁎
Retired (%)	.03	.15	.04	.23	.19	1.41
Disabled (%)	−.42	.19	5.15⁎	−.27	.26	1.13
Other (%)	−.18	.14	1.66	.37	.17	4.88⁎
Household size	−.05	.03	2.36	−.00	.05	.01
Level of urbanization^b^	−.03	.03	.81	−.11	.05	5.69⁎

a 0 = not yet attending any education; special (low-level) education; other sort of education/training/apprenticeship, 1 = kindergarten/primary, 2 = continued primary education or elementary secondary education, 3 = continued special (low-level) education, 4 = pre-university education, 5 = junior vocational training, 6 = senior vocational training, 7 = vocational colleges, 8 = vocational colleges 2nd tier, and 9 = university education.

b From 1 = very high degree of urbanization to 5 = very low degree of urbanization.

⁎⁎ p < 0.01.

⁎ p < 0.05.

### 3.3 Consideration of Future Consequences Scale scores and smoking levels

There was a negative association between CFCS scores and smoking status (r = −.09, p < .01), as shown in Table 2. Participants with a greater consideration of the future were less likely to identify themselves as smokers (*b* = −.01, SE = .01, χ^2^ = 7.05, p < .01), after controlling for demographic and background characteristics, as outlined in Table 4. The CFCS was not associated with heavy smoking levels in correlational (r = −.05, p = .11) or multivariate analyses (*b* = −.01, SE = .01, χ^2^ = 1.05, p = .31).

**Table 4:** Results of generalized estimating equations models assessing the relationship between consideration of future consequences scores and both smoking status and high cigarette consumption (N = 1218).

Variable	Smoking status			High cigarette consumption		
	B	SE	χ^2^	B	SE	χ^2^
Year	−.03	.01	22.39⁎⁎	−.03	.01	9.68⁎⁎
Consideration of Future Consequences Scale (CFCS)	−.01	.01	7.05⁎⁎	−.01	.01	1.05
Age	−.01	.00	6.27⁎	−.01	.00	2.86
Female	−.04	.09	.16	−.09	.12	.62
Education level completed^a^	−.05	.02	9.87⁎⁎	−.07	.02	13.86⁎⁎
Income	.00	.00	.88	.00	.00	1.32
Unemployed (%)	.38	.32	1.40	.67	.39	3.05
Retired (%)	−.07	.14	.25	.31	.17	3.34
Disabled (%)	−.27	.16	2.79	−.23	.22	1.15
Other (%)	.15	.13	1.34	.63	.15	16.97⁎⁎
Household size	−.02	.03	.51	.02	.04	.19
Level of urbanization^b^	−.06	.03	3.62	−.12	.04	8.38⁎⁎

a 0 = not yet attending any education; special (low-level) education; other sort of education/training/ apprenticeship, 1 = kindergarten/primary, 2 = continued primary education or elementary secondary education, 3 = continued special (low-level) education, 4 = pre-university education, 5 = junior vocational training, 6 = senior vocational training, 7 = vocational colleges, 8 = vocational colleges 2nd tier, and 9 = university education.

b From 1 = very high degree of urbanization, to 5 = very low degree of urbanization.

⁎⁎ p < 0.01.

⁎ p < 0.05.

### 3.4 Tobacco control measures and personality

Firstly, we used GEE analyses to test *model 2* and show that in the period after the introduction of smoking restrictions there was a reduction in heavy smoking (smoking 20 + cigarettes per day) (*b* = −.20, SE = .05, χ^2^ = 13.75, p < .01), over and above demographic and background factors and the time trend in smoking from 2001 to 2007. There was no evidence of such a decline in the prevalence of smoking more generally after the introduction of the tobacco control measures (*b* = −.04, SE = .03, χ^2^ = 1.27, p = .26).

Our test of *model 3* demonstrated a significant interaction between tobacco restrictions in 2004 and trait levels of self-control (*b* = .09, SE = .04, χ^2^ = 6.82, p < .01), as shown in Table 5. To understand the interaction effect we examined how the change in smoking levels after the introduction of the tobacco restrictions differed amongst those with low (24.1%), medium (29.9%), and high (46%) self-control. Subsequent analyses indicated that those with low self-control experienced a pronounced drop in heavy smoking after the introduction of tobacco control measures (*b* = −.30, SE = .10, χ^2^ = 8.67, p < .01), whereas those with high self-control showed no such decline (*b* = −.05, SE = .06, χ^2^ = .66, p = .42). CFCS scores did not interact with smoking regulations to predict changes in heavy smoking, as detailed in Table 6. As tobacco control measures did not appear to produce a reduction in the prevalence of smoking it was unsurprising that neither self-control nor CFCS scores modified the impact of the introduction of these regulations, as shown in Table 5, Table 6.

**Table 5:** Summary of generalized estimating equations models assessing the interaction between smoking regulations and self-control as a predictor of smoking status and high cigarette consumption (N = 1060).

Variable	Smoking status			High cigarette consumption		
	B	SE	χ^2^	B	SE	χ^2^
Year	−.02	.01	5.36⁎	.01	.01	.71
Self-control	−.14	.04	13.22⁎⁎	−.12	.05	7.14⁎⁎
Post-smoking regulation period	−.12	.11	1.15	−.67	.20	11.55⁎⁎
Self-control × post-smoking regulation period	.02	.02	.62	.09	.04	6.82⁎⁎

⁎⁎ p < 0.01.

⁎ p < 0.05.

**Table 6:** Summary of generalized estimating equations models assessing the interaction between smoking regulations and consideration of future consequences scores as a predictor of smoking status and high cigarette consumption (N = 1218).

Variable	Smoking status			High cigarette consumption		
	B	SE	χ^2^	B	SE	χ^2^
Year	−.02	.01	9.39⁎⁎	.00	.00	1.75
CFCS	−.02	.01	7.52⁎⁎	−.00	.00	−.21
Post-smoking regulation period	−.20	.18	1.36	−.04	.02	− 2.30⁎
CFCS × post-smoking regulation period	.00	.00	1.20	.00	.00	.82

⁎⁎ p < 0.01.

⁎ p < 0.05.

**Fig. 1:**
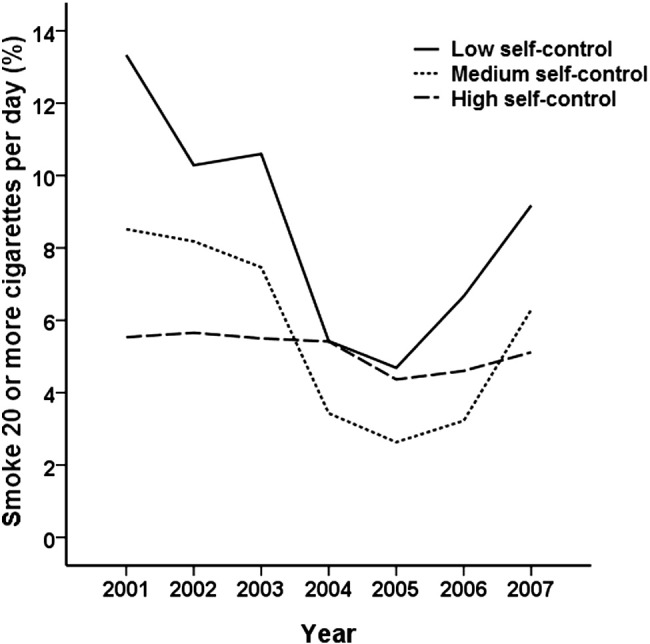
Heavy smoking levels (> 20 cigarettes per day) amongst participants from 2001 to 2007 as a function of low (24.1% of sample), medium (29.9%), and high (46%) self-control levels assessed in 2001. *Note*: The 2004 workplace smoking ban and tax increase on tobacco were introduced in the period between the collection of the 2003 and 2004 survey waves.

In order to test the impact of attrition in the sample over time on the pattern of results observed we repeated our analyses examining the interaction between trait self-control and tobacco restrictions amongst those who had observations on at least four of the seven years over the study period (N = 610). This portion of the sample had a high number of observations (M = 6.2, SD = 1.1) with almost 90% of observations present across the seven years examined (3805 of a potential 4270 observations). Visual inspection of the graphical trend in heavy smoking amongst those with low, medium, and high self-control revealed a pattern close to identical to that in Fig. 1 in this portion of the sample. The interaction effect was robust to this restriction and of the same magnitude as the effect in the full sample (*b* = .09, SE = .04, χ^2^ = 6.08, p < .05), suggesting that attrition is unlikely to have affected the interaction coefficient observed in the main analyses. As in the main analyses, the interaction in this portion of the sample with a high number of observations showed that those with low self-control experienced a large drop in heavy smoking after the introduction of tobacco control measures (*b* = −.35, SE = .11, χ^2^ = 9.79, p < .01), whereas those with high self-control showed no evidence of such a decrease (*b* = −.06, SE = .08, χ^2^ = .61, p = .43).

As a final robustness test we evaluate the potential role of socioeconomic status in explaining the interaction between self-control and tobacco restrictions in predicting changes in heavy smoking. It is possible that self-control may be acting as a proxy for low socioeconomic status and material resources which may explain a decline in heavy smoking in the aftermath of a large tax increase. However, including the interaction between tobacco restrictions and education and income either individually or in combination in the analyses failed to explain the pattern of results observed (self-control*tobacco restrictions coefficient in fully adjusted model including education*tobacco restrictions and income*tobacco restrictions terms alongside main effects: b = .095, SE = .036, χ2 = 7.08, p < .01). We interpret these tests as evidence against confounding by socioeconomic status.

### 3.5 Tobacco control measures, personality, and reuptake of heavy smoking

The change in heavy smoking levels from before to after the introduction of smoking restrictions for each self-control tertile is illustrated in Fig. 1. It is evident from Fig. 1 that those with low self-control showed a rapid decline in heavy smoking after the introduction of the 2004 smoking measures. However, the graph also points to a reuptake of heavy smoking in 2006 and 2007 amongst those with poor self-control, suggesting the benefits of the 2004 tobacco control measures may have been short-lived. We tested whether the non-linear U-shaped pattern of change in heavy smoking evident amongst those with low or medium self-control was statistically significant. Our analyses revealed a significant quadratic U-shaped trend in heavy smoking over the study period (b = .011, SE = .005, χ2 = 5.99, p < .05) which was present amongst those with low (b = .021, SE = .008, χ2 = 7.47, p < .01) and medium self-control (b = .020, SE = .010, χ2 = 4.05, p < .05) but not those with high self-control (b = −.001, SE = .007, χ2 = .02, p = .89). These results confirm the pattern of change evident in Fig. 1.

To further explore the evidence of reuptake of heavy smoking identified in Fig. 1 and the above analyses we also tested whether there was evidence that the interaction between self-control and tobacco restrictions diminished over time. These analyses showed that in the first year after the introduction of the tobacco restrictions the interaction effect of self-control and restrictions (coded so that 0 = 2001–2003, 1 = 2004) was strongest (b = .12, SE = .05, χ2 = 6.76, p < .01). When observed over progressively longer periods (i.e. tobacco restrictions coded so that: (i) 0 = 2001–2003, 1 = 2004–2005, (ii) 0 = 2001–2003, 1 = 2004–2006, (iii) 0 = 2001–2003, 1 = 2004–2007) there was evidence of a decline in the strength of the interaction effect by approximately a quarter (post-period is 2004-2005: b = .11, SE = .04, χ2 = 6.31, p < .05; post-period is 2004-2006: b = .10, SE = .04, χ2 = 6.87, p < .01; post-period is 2004-2007: b = .09, SE = .04, χ2 = 6.82, p < .01). Further post-hoc tests showed that the decline in the strength of the interaction coefficient over time verified the descriptive trend showing a gradual erosion of the tobacco restriction-linked heavy smoking reductions amongst those with low and medium self-control.

## 4 Discussion

Amongst the cohort of Dutch adults examined, those with greater self-control and future time perspective at baseline tended to show low levels of smoking over the seven year period of the study. These results complement prior findings (e.g. Adams and Nettle, 2009, Daly et al., 2014, Moffitt et al., 2011) suggesting that the psychological capacity for self-control and the tendency to consider future outcomes are predictive of lower smoking levels. Furthermore, our findings demonstrate a particularly close link between self-control and a reduced likelihood of heavy smoking (> 20 cigarettes per day) suggesting that self-control is not only related to smoking but may also shape the level of tobacco consumption amongst smokers.

Like many countries, the Netherlands introduced a legal prohibition on smoking in the workplace in recent decades. The effects of such laws are an important question for policymakers and researchers. As previously, we found that the introduction of a workplace smoking ban and a large tax increase on tobacco in the Netherlands in early 2004 appeared to have a beneficial effect on smoking levels (de Korte-de Boer et al., 2012, Verdonk-Kleinjan et al., 2009). We found that the tobacco control measures did not change the prevalence of smoking but were linked to a reduction in rates of heavy smoking, albeit only somewhat temporarily. More importantly, we found that personality assessed prior to the introduction of these major tobacco control measures modified the change in heavy smoking from before to after the introduction of the laws. People with high self-control showed no significant change in smoking patterns as a result of the laws. In contrast, rates of heavy smoking dropped substantially amongst people low in self-control, matching the levels of those with high self-control in the year after the smoking restrictions were introduced.

In the final year of our study, over three years after the ban took force, levels of heavy smoking showed an upturn, which again was most pronounced amongst people with low self-control. Rates of heavy smoking amongst people with low self-control regained most of what they had lost following the new law, though they did not reach the levels we found in the first year of our data (2001). The selective impact of the changes in tobacco policy on people with low self-control can be explained in at least two ways. One is that many of these people would like to quit smoking but do not have the inner discipline to do so. Hence they are aided by price increases and external restrictions (Gruber and Mullainathan, 2005, Hersch, 2005). Ample evidence indicates that smokers' cravings and lapses during quit attempts are affected by whether the environment permits or prohibits smoking (e.g. Carter and Tiffany, 2001, Juliano and Brandon, 1998). Indeed, the very desire to smoke seems to subside when it is explicitly forbidden. Hence people with low self-control might gain most by these effects of laws such as a workplace ban.

The second explanation is not that the workplace ban bolsters self-control but instead imposes burdens on it. When smoking is permitted, it is easily possible for a worker to sit at his or her desk and chain-smoke all day. Once the smoking ban takes effect, considerably more restraint and planning are required in order to continue smoking: For example, a worker must find a stopping point in the task and/or break in work, take the cigarettes outside or to a designated smoking area, consume the cigarette, and then return. Going outside requires dealing with the swiftly mutable Dutch weather. Schedules (e.g., planned meetings) must be respected and planned around. From this perspective, it is possible that people with low self-control reduced their smoking because they were relatively unwilling or unable to carry out this complex set of controlled actions (Fujita, 2011, Hofmann et al., 2012).

Although our data were not able to illuminate inner processes so as to distinguish between these two explanations, we think the weight of evidence favors the latter, though we do note that both theories could be correct to some degree. The fact that the tobacco control measures did not produce a reduction in all smoking, but only in heavy smoking, argues against the notion that people used the laws to help them quit. Rather, they seem to have cut down their smoking, which may have transformed many heavy smokers into light smokers (insofar as their habit dropped below 20 cigarettes per day). Moreover, the rebound in heavy smoking could indicate that after a couple years, even smokers with low self-control figured out how to smoke during working hours (possibly even drawing on the strategies of coworkers with high self-control) despite the restrictive laws. Alternatively, however, it could mean that smokers with low self-control did use the laws to reduce their smoking but then simply gave up, became accustomed to the increased prices, and resumed their previous level of smoking.

Some limitations of our study must be noted. The reliance on a single-item measure of self-control sacrifices measurement reliability and statistical power, so our results may underestimate the true power of the relationship between self-control and smoking and the impact of the tobacco control measures. This potential issue is evident in the study correlation matrix where weak associations were observed between self-control and the study covariates, several of which have been shown to be closely linked to self-control (e.g. Moffitt et al., 2011). We utilize self-reported data for the assessment of both personality and smoking. Future research may profitably incorporate fuller, more valid measures of self-control (e.g., Tangney, Baumeister, & Boone, 2004) alongside behavioral measures of inhibitory control and cotinine verified smoking levels. We also relied on an annual assessment of smoking which may explain why a decline in smoking prevalence was not identified in the aftermath of the 2004 smoking restrictions in this study (unlike prior studies where high frequency measurement of smoking was employed; Nagelhout et al., 2011). It is also important to note the workplace ban and 20% tax increase in 2004 were not the only anti-smoking measures in place in the Netherlands at this time (see Verdonk-Kleinjan et al., 2011). Less intensive tobacco control measures were implemented in the period prior to and after 2004. Cigarette advertising was restricted in 2002 and youth access to tobacco was restricted in 2003. Minimum cigarette price laws and additional taxes on tobacco went into effect in 2005, increasing prices by an average of 4%. As a result of these laws and perhaps a growing public awareness of the dangers of tobacco, smoking rates declined over a multi-year period. Still, such effects would work against our ability to detect specific effects of the 2004 tobacco control measures, so it is all the more striking that significant reductions occurred specifically in response to the new laws.

Further studies are needed to precisely identify how self-control interacts with specific attributes of national tobacco control policy (Amador and Nicolás, 2013, Hersch, 2005). Embedding self-control measures into experimental or quasi-experimental studies will shed new light on heterogeneity in the price sensitivity of smoking and the reaction of smokers to environmental restrictions. The differential effect of tobacco control measures on people with differing levels of self-control has major implications for how policies could be targeted to enable people with self-control deficits to improve their own health behavior. This potentially fruitful area of research would require micro-data across countries or states that permit natural policy experimentation and also include information on self-control.

## 5 Conclusions

This study combined individual-level psychological and behavioral measures to examine the impact of a major national tobacco reform. Our findings add to the growing body of research demonstrating the value of self-control for promoting consequential heath behaviors and important outcomes (e.g. Daly et al., 2015, Moffitt et al., 2011). Our data indicated that self-control is linked to a reduced likelihood of smoking and may also shape how tobacco restrictions influence heavy smoking levels. We showed that the introduction of major tobacco control measures in the Netherlands in 2004 was linked to a reduction in the number of people smoking twenty or more cigarettes per day, and this effect was mainly found amongst smokers with low self-control. The tobacco restrictions do not appear to have helped people quit smoking altogether, but rather helped these smokers cut down, switching from being heavy smokers to moderate or light smokers. The effect appears to have lasted for a couple of years but then largely dissipated, as rates of heavy smoking amongst people low in self-control rose again. As many individuals, therapists, and other groups can affirm, the battle against smoking has at least two major phases, one instigating the initial reduction or quitting, and the second maintaining the abstinence. Legal restrictions appear to help with the first phase, but their long-term utility for the second phase requires further research.

## Funding sources

We gratefully acknowledge funding support from the Economic and Social Research Council (ES/L010437/1). However, the Research Council bears no responsibility for the study design, analysis or interpretation of these data, writing the manuscript, or the decision to submit the paper for publication.

## Contributors

M. Daly and L. Delaney developed the study concept. M. Daly performed the statistical analyses. M. Daly, Delaney and R.F. Baumeister drafted the manuscript and provided critical revisions. All authors approved the final version of the manuscript for submission.

## Conflict of interest

All authors declare that they have no conflict of interest.
